# Safety and Efficacy of Allogeneic Natural Killer Cells in Combination with Pembrolizumab in Patients with Chemotherapy-Refractory Biliary Tract Cancer: A Multicenter Open-Label Phase 1/2a Trial

**DOI:** 10.3390/cancers14174229

**Published:** 2022-08-30

**Authors:** Galam Leem, Sung-Ill Jang, Jae-Hee Cho, Jung Hyun Jo, Hee Seung Lee, Moon Jae Chung, Jeong Youp Park, Seungmin Bang, Da-Kyung Yoo, Hyo-Cheon Cheon, Jae-Eun Kim, Kyeong-Pill Lim, In-Hye Jung, Jung-Min Im, Yong-Yoon Chung, Seung Woo Park

**Affiliations:** 1 Division of Gastroenterology, Department of Internal Medicine, Severance Hospital, Yonsei University College of Medicine, Seoul 03722, Korea; 2 Department of Internal Medicine, Gangnam Severance Hospital, Yonsei University College of Medicine, Seoul 06273, Korea; 3 Research Institute of Cell Biology, SMT Bio Co., Ltd., Seoul 08503, Korea

**Keywords:** allogeneic natural killer cell therapy, pembrolizumab, immunotherapy, chemotherapy-refractory advanced biliary tract cancer

## Abstract

**Simple Summary:**

Since there is no effective subsequent-line therapy for patients with cisplatin/gemcitabine-refractory advanced biliary tract cancer, there is an urgent need for new treatment options. This phase 1 and 2a study is the first clinical trial reporting the safety and the efficacy of allogeneic NK cells (“SMT-NK”) in combination with pembrolizumab in patients with chemotherapy-refractory advanced biliary tract cancer. We found that the SMT-NK can be safely administered with pembrolizumab to patients with the chemotherapy-refractory advanced biliary tract cancer, and the combinational therapy of SMT-NK and pembrolizumab showed improved efficacy outcome compared to recent trials using pembrolizumab alone. This study provides evidence for a new possible treatment option for advanced biliary tract cancer patients.

**Abstract:**

Background and Aim: This study investigated the administration of combination therapy, allogeneic natural killer (NK) cells and pembrolizumab in the treatment of advanced biliary tract cancer to determine the safety and tolerability (phase 1) and the efficacy and safety (phase 2a). Methods: Forty patients (phase 1, *n* = 6; phase 2a, *n* = 34) were enrolled between December 2019 and June 2021. The patients received highly activated allogeneic NK cells (“SMT-NK”) on weeks 1 and 2 and pembrolizumab on week 1. This 3-week schedule (one cycle) was repeated until confirmed disease progression, intolerable adverse events (AEs), patient withdrawal, or finishing the maximum treatment schedule. The tumor response was evaluated after every three cycles. Results: In phase 1, four patients (66.7%) experienced seven AEs, but no severe AE was observed. In phase 2a, 126 AEs occurred in 29 patients (85.3%). Severe AEs (≥grade 3) were reported in 16 patients (47.1%). The overall response rate (ORR) was 17.4% in the full analysis set and 50.0% in the per-protocol set. Conclusions: SMT-NKs plus pembrolizumab resulted in no severe AEs directly related to the drug combination. The combination therapy also exerted antitumor activity with improved efficacy compared to the recent monotherapy with pembrolizumab in patients with advanced biliary tract cancer.

## 1. Introduction

Biliary tract cancers, including cholangiocarcinoma (cancers arising in the intrahepatic, perihilar, or extrahepatic bile duct) and gallbladder carcinoma, are some of the most incurable cancers [1]. As biliary tract cancers are usually asymptomatic during the early stages, more than 50% of patients with these cancers are diagnosed in the advanced stages [2,3]. For the primary treatment of unresectable and metastatic biliary tract cancers, systemic chemotherapy is the standard therapy, and cisplatin plus gemcitabine is the preferred regimen as the first-line chemotherapy [4,5]. However, the overall survival has been reported to be less than 1 year. Furthermore, effective subsequent-line therapies for cisplatin/gemcitabine-refractory patients are currently limited, resulting in a poor prognosis for advanced biliary tract cancer patients [6,7,8]. Therefore, there is an urgent need to broaden the treatment options.

Immunotherapy can be an option for treating advanced biliary tract cancers based on the remarkable success of using immune checkpoint inhibitors (ICIs), including blocking antibodies against programmed cell death-1 (PD-1)/programmed cell death ligand-1 (PD-L1) and cytotoxic T-lymphocyte-associated antigen 4 (CTLA-4), in treating patients with several cancers [9,10,11]. Pembrolizumab, a PD-1 inhibitor, has replaced conventional chemotherapy as the first-line treatment for patients with melanoma, non-small cell lung cancer, and renal cell carcinoma [12,13,14,15]. Several clinical trials using the PD-1 inhibitors pembrolizumab or nivolumab have also been conducted in patients with advanced biliary tract cancers [16,17,18,19]. Durable responses and improved survival have been found in some patients but only in the minority (objective response rate (ORR) 3–13%). A very recent study of pembrolizumab treatment alone had an ORR of 10%, with a median progression-free survival (PFS) of 1.5 months [19]. These studies suggest that there is a need for combinational therapy that overcomes the limited efficacy of the monotherapies.

Natural killer (NK) cells are a major subset of innate lymphocytes and play an important role in protecting against viral infection or cancer cells [20,21]. NK cells recognize and destroy cancer cells that lack major histocompatibility complex (MHC) class I, which interfere with the cytolytic function of NK cells by ligation to killer cell immunoglobulin-like receptors (KIRs) expressed on NK cells [22,23]. NK cells can be categorized into two subsets based on the relative surface expression of CD56 and CD16: the cytolytic CD56^dim^CD16^pos^ NK cell subset and the cytokine-producing CD56^bright^CD16^neg^ NK cell subset [24,25]. Clinical trials have been performed using healthy donor-derived allogeneic NK cells in various cancer patients [26,27], and the possibility of combinational therapy with allogeneic NK cells and pembrolizumab is emerging [28]. In a previous study, we reported that NK cells have a beneficial effect on controlling cholangiocarcinoma in mice in vivo [29]. However, no studies have reported the efficacy of NK cell therapy in patients with biliary tract cancer.

We designed a multicenter open-label phase 1/2a clinical trial to assess the safety and efficacy of allogeneic NK cells (“SMT-NK”) in combination with pembrolizumab in patients with conventional chemotherapy-refractory biliary tract cancer.

## 2. Materials and Methods

### 2.1. Study Design and Participants

Patients aged ≥ 19 years with histologically confirmed unresectable advanced biliary tract cancer who progressed after at least one regimen of chemotherapy or were unable to proceed with chemotherapy due to significant side effects were enrolled in this study at two individual health centers: Yonsei University Severance Hospital (Seoul, Korea) and Gangnam Severance Hospital (Seoul, Korea). Additionally eligible for enrollment were patients with a combined positive score (CPS) for the tumor > 1%, positively determined microsatellite instability–high (MSI-H), or deficient mismatch repair (dMMR). The CPS was calculated by dividing the number of PD-L1-positive tumor cells, lymphocytes, and macrophages by the total number of viable tumor cells and multiplying by 100. The MSI-H was determined by polymerase chain reaction (PCR) and considered positive when two or more unstable markers were found among five microsatellite markers. The dMMR was analyzed by immunohistochemical staining and considered positive when one or more genes were lost when staining for MLH1, MSH2, MSH6, and PMS2. Patients were ineligible if they had a history of any immunodeficiency disease, immunotherapy, or other site tumors within 5 years before enrollment in this study. A full list of the inclusion and exclusion criteria is presented in Table S1.

The primary objectives of the phase 1 trial were to determine the safety, including adverse events (AEs), adverse drug reactions (ADRs), laboratory tests, vital signs, and Eastern Cooperative Oncology Group (ECOG) performance status, and the tolerability by dose-limiting toxicity (DLT) of the combination of allogeneic NK cells and pembrolizumab. The primary objective of the phase 2a trial was the ORR (complete response (CR) and partial response (PR)) at the end of the study protocol. The secondary objectives were the disease control rate (DCR), time to progression (TTP), PFS, and DLT at the end of the study protocol. The severity of the AEs and ADRs were classified using common terminology criteria for adverse events (CTCAE, version 5.0). The DLT was evaluated based on National Cancer Institute-common toxicity criteria (NCI-CTC) version 4.03.

All patients provided written informed consent. This study was conducted in accordance with the Declaration of Helsinki (1996) and approved by the institutional review boards of all the participating institutions.

This study is registered with ClinicalTrials.gov (number NCT03937895).

### 2.2. Study Protocol

During the 3-week treatment schedule, which defined one cycle, patients intravenously received highly activated allogeneic NK cells (“SMT-NK”, 3 × 10^6^ NK cells per kg) on weeks 1 and 2 and received pembrolizumab (Keytruda^®^) 200 mg prior to NK cell injection on week 1 (Figure S1). On week 3, patients took a rest without treatment. The treatment cycle was repeated until the end of the trial, with a total of 3 and 9 cycles in phases 1 and 2a, respectively. Finishing all of the phase 1 schedule, the patients with a stable disease (SD) or higher efficacy status and without DLT were enrolled in the phase 2a trial and continued the treatment schedule. The efficacy of the treatment was monitored by computed tomography (CT) after every three cycles, which was compared to the baseline values based on the Response Evaluation Criteria in Solid Tumors (RECIST) version 1.1. Patients interpreted as having a progressive disease (PD) in the efficacy evaluation did not continue with further cycles in the trial, and if they were interpreted as having SD, PR, or CR, they proceeded up to 9 cycles, and up to three efficacy evaluations were conducted.

### 2.3. Preparation and Ex Vivo Expansion of Allogeneic NK Cells

At least 2 weeks before each injection of SMT-NKs, 60–80 mL of whole blood was harvested from a healthy donor who did not meet any exclusion criteria (Table S2). We isolated peripheral blood mononuclear cells (PBMCs) from the whole blood and depleted CD3^+^ T cells using MACSxpress (Miltenyi Biotec, Bergisch Gladbach, Germany). The T-cell-depleted PBMCs were then washed two times with Dulbecco’s phosphate-buffered saline (DPBS) and cultured in a T75 flask containing 20 mL of NK expansion medium (ALyS505NK-IL2 1000 IU/mL, Cell Science & Technology Institute, Sendai, Japan). The IL-2-activated NK cells were fed fresh medium every 3 days and transferred to a T175 flask after 5–7 days of culture. The NK cell expansion was continued for another 7–14 days by adding fresh medium until a desired cell number was reached. The viability and number of expanded NK cells was measured by a trypan blue counting method with an automatic cell counter.

### 2.4. Characterization of NK Cells

To analyze the NK cell population, the obtained PBMCs were stained with PerCP-CyTM5.5-conjugated anti-human CD56 and FITC-conjugated anti-human CD3 antibodies (BD Biosciences, San Jose, CA, USA). The cells were washed twice with phosphate-buffered saline (PBS) and resuspended in FACS-staining buffer (PBS with 0.5% bovine serum albumin). The cells were then stained with the appropriate antibodies at 4 °C for 30 min. After washing with PBS three times, the cells were analyzed with a flow cytometer (CytoFLEX).

The cytolytic NK cell activity was measured using Cell Counting Kit-8 (CCK-8; Dojindo Molecular Technologies, Rockville, MD, USA). K562 human leukemia cells were included as positive target cells to compare the cytolytic activity of the NK cells against HuCCT-1 human cholangiocarcinoma cells in the cytotoxicity assay. K562 cells and HuCCT-1 cells were cocultured with the isolated NK cells (T-cell-depleted PBMCs as described above) at an effector-to-target cell ratio of 5:1 in 96-well plates. After 24 h, the viability of the target cells was measured by CCK8 following the manufacturer’s instructions. Absorbance was measured at 450 nm using a microplate reader.

The interferon-γ (IFN-γ) concentration was measured in the culture supernatants of isolated NK cells using the human IFN-γ Pre-Coated ELISA kit (BioGems, Westlake Village, CA, USA) in accordance with the manufacturer’s protocol. The absorbance at 450 nm was measured in each well using a microplate reader (ELx808).

### 2.5. Statistical Analysis

The data were analyzed as a full analysis set, per-protocol set, and safety set according for the clinical trial protocol. For the safety evaluations in phases 1 and 2a, the safety set was used. For the efficacy evaluation in phase 2a, the full analysis set was used as the main analysis group and the per-protocol set as the auxiliary.

The ORR was defined as the ratio of patients whose tumor evaluation was PR or CR. The radiological responses were categorized per RECIST version 1.1 and the best response reported. The number and ratio of patients for the primary efficacy rate (i.e., ORR) were presented, with a 95% confidence interval (CI) for the ratio. For TTP, the survival function was estimated and presented by the Kaplan–Meier method. The TTP was defined as the time from first pembrolizumab administration to the time at which the disease was confirmed as progressive or the last observation time. Patients who did not progress at the time of completion of the clinical trial or were lost to follow-up due to dropout (including those who died before disease progression) were censored at the time of the last tumor evaluation date.

Toxicity was evaluated as the occurrence of AEs corresponding to the DLT. The PFS and overall survival period (OS) were also considered as exploratory efficacy evaluation variables. For each variable, the survival function was estimated by the Kaplan–Meier method, and the median value of each variable with the 95% CI for both sides was presented.

In this clinical trial, unless otherwise specified, the significance level was 5% on both sides, or a 95% CI was presented. All statistical analyses were performed using well-known and validated statistical software SAS (Ver. 9.4; SAS Institute, Cary, NC, USA).

## 3. Results

### 3.1. Patient Characteristics

**Figure 1:**
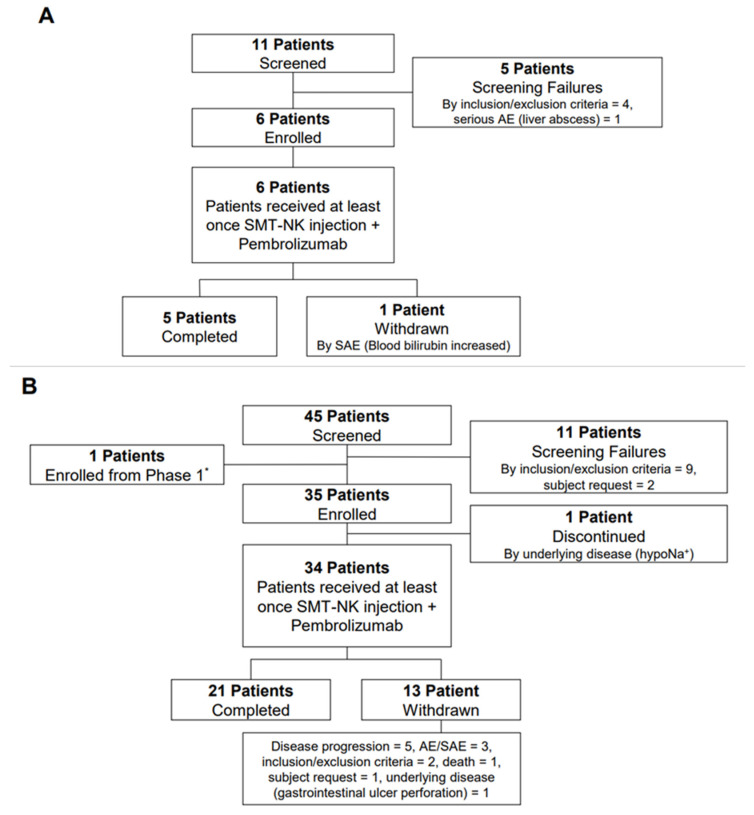
Trial profile. (**A**), phase I; (**B**), phase IIa. * One patient showed a stable disease after completion of the phase 1 trial and enrolled in the phase 2a trial. AE, adverse event; SAE, serious adverse event.

Between December 2019 and June 2021, 56 patients were screened for enrollment (11 for the phase 1 trial and 45 for the phase 2a trial). Six eligible patients were enrolled in the phase 1 trial, and five of them completed the study. One of these patients had a stable response to the treatment and proceeded into the phase 2a trial. Thirty-four eligible patients plus one patient from the phase 1a trial were enrolled in the phase 2a trial (Figure 1). Among the patients in the phase 2a trial, the patients who received SMT-NKs and pembrolizumab at least once were defined as the safety set (*N* = 34). Among the patients who received SMT-NKs and pembrolizumab at least once, the patients for whom we could obtain data on the efficacy evaluation were defined as the full analysis set (*N* = 23), and the patients who completed the study protocol were defined as the per-protocol set (*N* = 8). The patient demographics and baseline characteristics are presented in Table S3. All enrolled patients had previously received chemotherapy, and most of the chemotherapy regimens were gemcitabine-based (85.5% and 100% for the phase 1 trial and phase 2a trial, respectively).

### 3.2. Tumor Response

**Figure 2:**
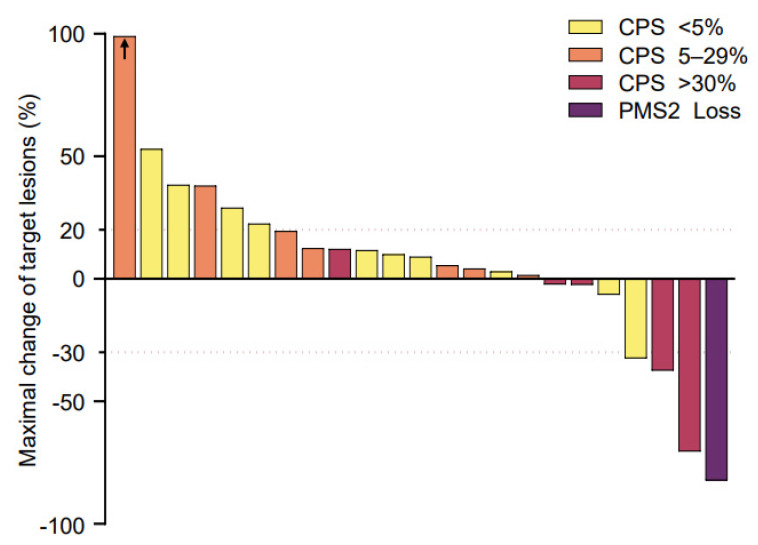
Best changes in the sizes of the target lesions from the baseline for 23 patients in the phase 2a trial. The change in target lesions was calculated as the ratio to the baseline diameters measured in computed tomography (CT) images. The yellow bars indicate patients with a combined positive score (CPS) < 5%, orange bars CPS 5–29%, and red bars CPS > 30%. The purple bar indicates a patient who lost *PMS2*. The arrow indicates that the patient had a 169% increase in the size of the target lesion according to Response Evaluation Criteria in Solid Tumors (RECIST) version 1.1, and of the scale for the figure is inaccurate. The upper red dashed line indicates a 20% increase in the size of the target lesion from the baseline, and the lower red dashed line indicates a 30% decrease in the size of the target lesion from the baseline.

**Figure 3:**
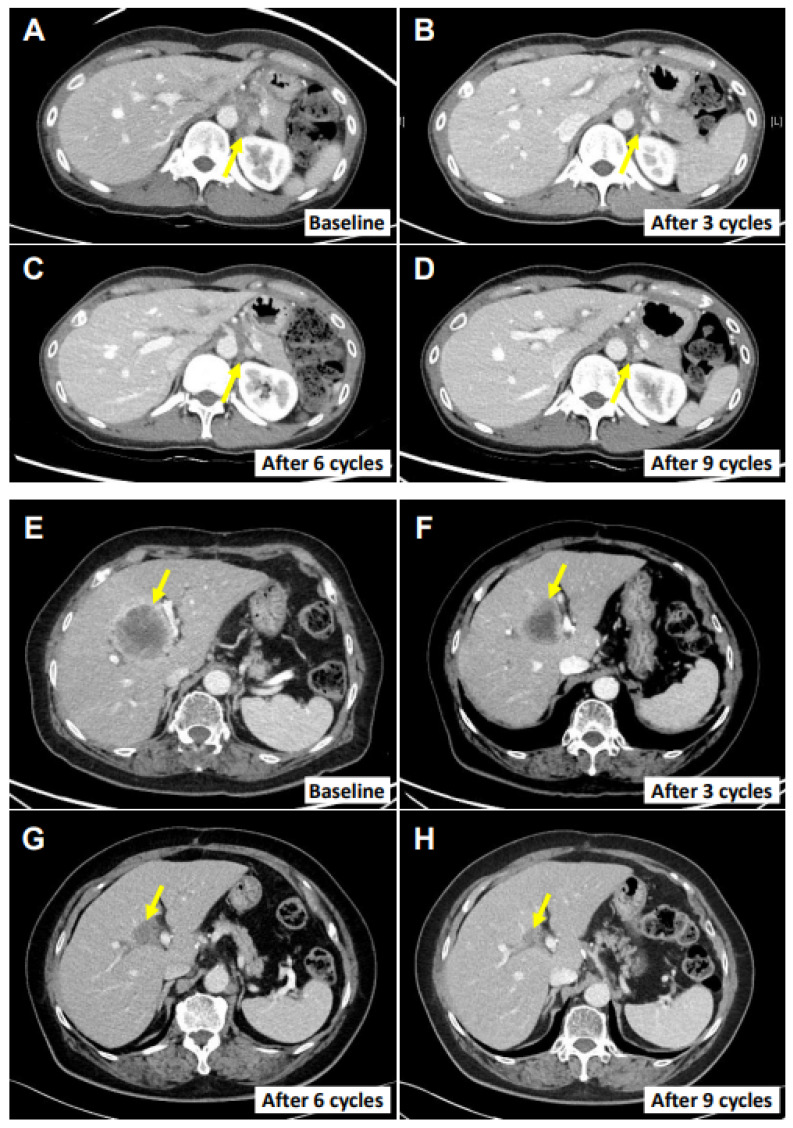
Cancer regression in two patients. CT images of the main tumor lesions at the baseline in each response evaluation. (**A**–**D**) Left infrarenal lymph node in patient E0107. (**E**–**H**) The main hepatic metastasis lesion in patient E0217. Yellow arrows indicate the target lesions. Response evaluations were performed after every three cycles.

**Figure 4:**
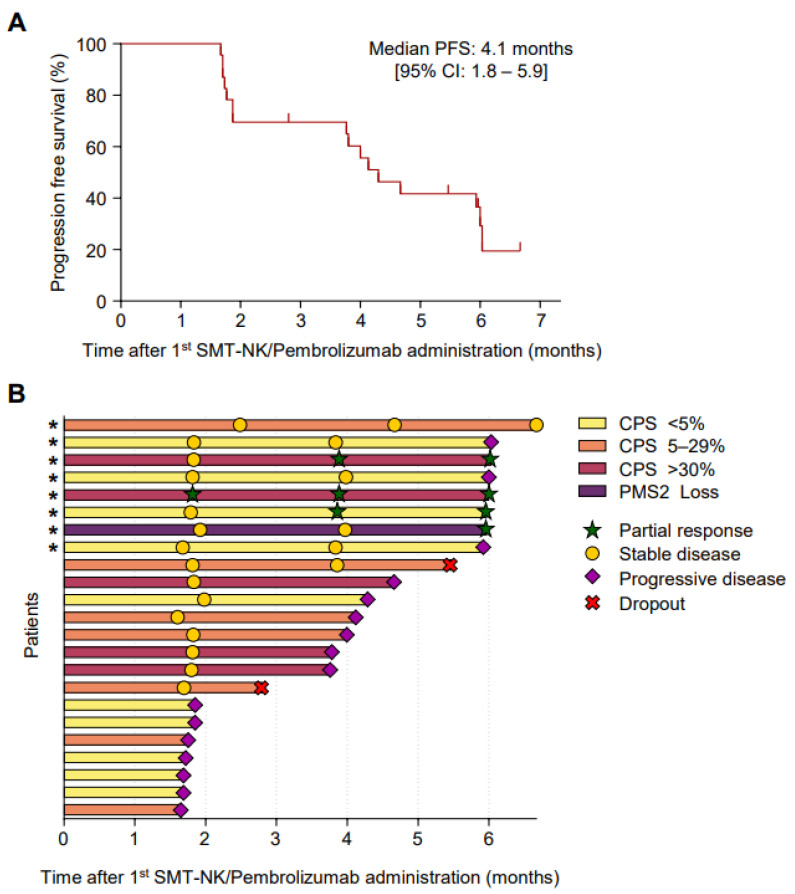
Progression-free survival and time to progression for each patient. (**A**) Kaplan–Meier estimates of the progression-free survival in the full analysis set. (**B**) Time to progression. The yellow bars indicate patients with a combined positive score (CPS) < 5%, orange bars CPS 5–29%, and red bars CPS > 30%. The purple bar indicates a patient who lost *PMS2*. * The patient completed 27 cycles of treatment.

The ORR (CR and PR) at the end of the study protocol was 17.4% (95% CI, 5.0–38.8%) in the full analysis set and 50.0% (95% CI, 15.7–84.3%) in the per-protocol set. The DCR (CR, PR, and SD) at the end of the study protocol was 30.4% (95% CI, 13.9–54.9%) in the full analysis set and 62.5% (95% CI, 24.5–91.5%) in the per-protocol set (Table 1). There was no significant difference in the ORR according to the CPS (Table S4). When considering the best responses of each patient, the DCR was up to 73.9%, and the maximal change in the targeted lesions from the baseline was −82.3% to 169% (Figure 2). Importantly, two patients demonstrated the sustained objective regression of their main targeted lesions, as assessed by RECIST. Patient E0107, a 40-year-old female diagnosed with gallbladder cancer with peritoneal carcinomatosis after undergoing cholecystectomy due to cholecystitis, had previously received chemotherapies including gemcitabine, cisplatin, 5-fluorouracil (5-FU), onivyde^®^ (irinotecan liposome), and leucovorin. After treatment with SMT-NKs and pembrolizumab as described above, she experienced an 82.3% reduction in the metastatic lymph nodes (Figure 3A–D) and remained progression-free 18 months after the initial treatment. Patient E0217, a 76-year-old female diagnosed with extrahepatic cholangiocarcinoma, underwent surgery for the segmental resection of the common bile duct and cholecystectomy. Four months later, several hepatic metastases were found, and she received systemic chemotherapy with gemcitabine and cisplatin. The multiple hepatic lesions then progressed. After the SMT-NK and pembrolizumab treatment as described above, she experienced a 70.4% reduction in the hepatic metastasis lesions (Figure 3E–H) and remained free from progression 12 months after the initial treatment. At the time of analysis, the median PFS was 4.1 months (95% CI, 1.8–5.9 months) in the full analysis set (Figure 4). As the progression rate was less than 50%, the median PFS in the per-protocol set could not be calculated. We found no significant difference in the PFS according to the CPS (Table S5). The mortality rate was less than 50% in both sets, so the median overall survival could not be estimated in any set.

### 3.3. Safety and Tolerability

Safety and tolerability were assessed in the 6 patients in phase 1 and 34 patients in phase 2a who received at least one dose of SMT-NKs and pembrolizumab from the day of the first administration to 12 weeks after the last administration of the clinical trial drugs. In the phase 1 trial, four patients (66.7%; 95% CI, 22.3–95.7%) experienced seven AEs (four grade 1 and three grade 3 events; Table 2). Three severe AEs (grade 3) occurred in one patient (16.7%; 95% CI, 0.4–64.1%), which was definitely not related to the administration of clinical trial drugs; however, due to the AEs, the further administration of SMT-NKs and pembrolizumab was postponed and discontinued. No DLT was reported.

In the phase 2a trial, 126 AEs occurred in 29 patients (85.3%; 95% CI, 68.9–95.1%). The most frequent events were grades 1 or 2 and consisted of pruritus (14.7%), abdominal pain (11.8%), fever (17.6%), general weakness (14.7%), and limb edema (8.8%; Table S6). Grades 3–5 AEs were reported in 16 patients (47.1%; 95% CI, 29.8–64.9%). Two patients (5.9%) decided to delay any further treatment cycles, and 12 patients (35.3%) decided to discontinue any further treatment cycles. Two patients experienced grade 5 AEs. Patient E0110 experienced grade 5 jaundice, which was not related to the clinical trial drugs, and discontinued any further cycles due to AE-related death. Patient E0215 was diagnosed with newly developed cholangiocarcinoma during the trial and decided to stop any further administration of the clinical trial drugs. No DLT was reported.

### 3.4. Biomarkers

To determine whether the patients’ tumor response depended on the characteristics of the NK cells, we performed a flow cytometric analysis using isolated NK cells from donor PBMCs. We analyzed the frequency of CD56^+^ cells among CD3^−^ cells and PD-1-positive cells and CD107a-positive cells among NK cells. We found no significant difference between the patient groups (Figure S2A). We also evaluated the function of NK cells. We measured the IFN-γ concentration using the culture supernatants of isolated NK cells and assessed the NK cell cytotoxicity against K562 cells and HuCCT-1 cells. We found no significant difference in the function of NK cells between patient groups (Figure S2A). We also analyzed the characteristics and function of the NK cells from the patients and found that the expression of PD-1 on NK cells was upregulated in patients with PD compared to the other outcomes (Figure S2B), suggesting that allogeneic NK cell therapy would be more beneficial to the patients whose peripheral NK cells have a weaker antitumor function [30,31]. No significant functional differences in the NK cells were found.

## 4. Discussion

To the best of our knowledge, this SMT-NK and pembrolizumab combination study is the first phases 1 and 2a trial to demonstrate the safety, tolerability, and efficacy of allogeneic NK cells in patients with chemotherapy-refractory biliary tract cancer. Notably, the administration of allogeneic NK cells in combination with pembrolizumab did not cause more AEs than pembrolizumab monotherapy, as previously reported in advanced biliary tract cancers [18,19,32]. Two patients experienced grade 5 AEs in this study (5.9%), but they were not related to the drugs. Furthermore, no DLT was reported. More importantly, the administration of SMT-NKs with pembrolizumab resulted in an increased ORR compared to previous clinical trials using pembrolizumab in patients with chemotherapy-refractory advanced biliary tract cancers [19,32]. Taken together, the results of this clinical trial provide evidence for future large, randomized trials comparing allogeneic NK cells and pembrolizumab to conventional chemotherapies for the treatment of chemotherapy-refractory advanced biliary tract cancer.

The administration of SMT-NKs in combination with pembrolizumab had a modest safety profile and was well-tolerated in this study. Four patients in the phase 1 trial and twenty-nine patients in the phase 2a trial suffered from an AE, and the most frequent events were consistent with previous studies of pembrolizumab monotherapy [16,17,19,32]. One patient in the phase 1 trial and twelve patients in the phase 2a trial discontinued the trial due to serious AEs (grades 3–5). The most common serious AEs were hematological toxicities and hepatobiliary toxicities. Despite the use of allogeneic NK cells derived from healthy donors, no serious graft-versus-host disease (GVHD) was reported, indicating that SMT-NKs did not induce any non-specific cytotoxicity against host tissues.

In this clinical trial, the ORR and DCR in the full analysis set were 17.4% (95% CI, 5.0–38.8%) and 30.4% (95% CI, 13.9–54.9%), respectively. When analyzing only patients who completed 27 cycles of the treatment, the ORR and DCR increased to 50.0% (95% CI, 15.7–84.3%) and 62.5% (95% CI, 24.5–91.5%), respectively. The median PFS in the full analysis set was 4.1 months (95% CI, 1.8–5.9 months). Considering that the enrolled patients in this clinical trial were resistant to prior conventional chemotherapies based on gemcitabine, these efficacy outcomes can be evaluated as very effective. Currently, 5-FU and oxaliplatin (FOLFOX), 5-FU and irinotecan (FOLFIRI), or regorafenib are preferred as second-line chemotherapy in advanced biliary tract cancers, but the ORR has not exceeded 11%, and the PFS has been reported to be less than 4 months [5,7,33,34,35]. To overcome this limitation of the current conventional chemotherapies, several clinical trials using pembrolizumab as a second-line or greater treatment have been conducted in patients with advanced biliary tract cancer. A recent Korean study of 40 advanced biliary tract cancer patients using pembrolizumab reported an ORR of 10% and median PFS of 1.5 months (95% CI, 0–3.0 months) [19]. The KEYNOTE-028 study of 24 patients reported an ORR of 13% (95% CI, 2.8–33.6%) and median PFS of 1.8 months (95% CI, 1.4–3.1 months). The KEYNOTE-158 of 104 patients reported an ORR of 5.8% (95% CI, 2.1–12.1%) and median PFS of 2.0 months (95% CI, 1.9–2.1 months) [32]. Comparing the clinical outcomes of previous clinical trials with our results, the administration of SMT-NK with pembrolizumab would be beneficial in patients with chemotherapy-refractory advanced biliary tract cancer. Further randomized clinical trials are required to compare the effects of the SMT-NKs and pembrolizumab to the current second-line conventional chemotherapies.

Given the heterogeneous nature and prognosis of biliary tract cancer, the small number of enrolled patients is a possible limitation of this study. Forty patients were sufficient to evaluate the safety, tolerability, and efficacy as initially planned, but it was insufficient to analyze the tumor locations. In addition, the median follow-up of 2.1 months (95% CI, 1.9–4.2 months) was not long enough to estimate the OS. Furthermore, as this study was a single-arm study, it was not possible to compare the effects of combinational therapy with SMT-NKs and pembrolizumab to SMT-NK monotherapy or pembrolizumab monotherapy. Therefore, this study was limited to determining whether the administration of SMT-NKs and pembrolizumab induced a synergistic effect or an additive effect. Considering that PD-1, which was originally described on T lymphocytes, is also expressed on NK cells [36,37,38], it is thought that the administration of SMT-NKs in combination with pembrolizumab can induce a synergistic effect on each other. Further clinical trials in additional patients are being planned to compare the effects of combinational therapy with SMT-NKs and pembrolizumab to pembrolizumab monotherapy.

## 5. Conclusions

In conclusion, to the best of our knowledge, this was the first clinical trial to report the safety and efficacy of allogeneic NK cells in combination with pembrolizumab in patients with advanced biliary tract cancer. We found that the SMT-NKs can be safely administered with pembrolizumab to patients with chemotherapy-refractory advanced biliary tract cancer, and the combinational therapy resulted in a higher response rate and longer PFS than previously reported for pembrolizumab monotherapy [18,19,32]. However, since this study was a nonrandomized, single-arm study, further large randomized phase 2/3 trials are recommended to confirm the effects of SMT-NKs in combination with pembrolizumab and to elucidate the role of SMT-NKs and pembrolizumab in the treatment of advanced biliary tract cancer.

## Figures and Tables

**Table 1:** Objective response rate and disease control rate.

	Full Analysis Set (*N* = 23)	Per-Protocol Set (*N* = 8)
Tumor response		
CR	0 (0.0)	0 (0.0)
PR	4 (17.4)	4 (50.0)
SD	3 (13.0)	1 (12.5)
PD	16 (69.6)	3 (37.5)
Objective response rate ^a^	4 (17.4)	4 (50.0)
(95% CI)	(5.0–38.8%)	(15.7–84.3%)
Disease control rate ^b^	7 (30.4)	5 (62.5)
(95% CI)	(13.9–54.9%)	(24.5–91.5%)

^a^ CR + PR and ^b^ CR + PR + SD. CR, complete response; PR, partial response; SD, stable disease; PD, progressive disease; CI, confidence interval. Data are presented as *n* (%) unless otherwise noted.

**Table 2:** Summary of the adverse events and adverse drug reactions.

	Phase 1 (*N* = 6)	Phase 2a (*N* = 34)
Patients with any AE	4 (66.7) (22.3–95.7%)	29 (85.3) (68.9–95.1%)
Number of events	7	126
Patient characteristics		
Patients with serious AE	1 (16.7) (0.4–64.1%)	16 (47.1) (29.8–64.9%)
Patients with ADR	2 (33.3) (4.3–77.7%)	7 (20.6) (8.7–37.9%)
Patients with serious ADR	0 (0.0)	1 (2.9) (0.1–15.3%)
Patients with unexpected ADR	0 (0.0)	0 (0.0)
Grade		
Grade 1/mild AE	4 (66.7)	23 (67.6)
Grade 2/moderate AE	0 (0.0)	18 (52.9)
Grade 3/severe AE	1 (16.7)	13 (38.2)
Grade 4/life-threatening or disabling AE	0 (0.0)	0 (0.0)
Grade 5/death related to AE	0 (0.0)	2 (5.9)
Outcome		
Recovered	3 (50.0)	24 (70.6)
Recovered with sequelae	0 (0.0)	2 (5.9)
Recovering	0 (0.0)	7 (20.6)
Not yet recovered	2 (33.3)	14 (41.2)
Lost to follow-up	0 (0.0)	0 (0.0)
Death	1 (16.7)	3 (8.8)
Action taken with NP		
Dose not changed	4 (66.7)	25 (73.5)
Dose delayed	1 (16.7)	5 (5.9)
IP withdrawn	1 (16.7)	12 (35.3)

AE, adverse event; ADR, adverse drug reaction; NP, injection of SMT-NKs and pembrolizumab. Data are presented as *n* (%) with or without a 95% confidence interval.

## Data Availability

All data obtained and analyzed for this clinical study are available from the corresponding author (S.W.P.) upon reasonable request.

## Supplementary Materials

The following supporting information can be downloaded at https://www.mdpi.com/article/10.3390/cancers14174229/s1. Figure S1: Study protocol for the phase 1 (A) and phase 2a (B) trials. Figure S2: The characteristics and functional activities of NK cells from donors (A) and recipients (B). Table S1. Inclusion and exclusion criteria for patient enrollment. Table S2. Inclusion and exclusion criteria for healthy NK cell donor. Table S3. Demographics and baseline characteristics of enrolled patients. Table S4. Objective Response Rate (ORR) by Combined Positive Score in Phase 2a. Table S5. Progression free survival (PFS) by Combined Positive Score in Phase 2a. Table S6. Adverse events by system organ class.
