# Supplementary material for: Safety and Efficacy of Allogeneic Natural Killer Cells in Combination with Pembrolizumab in Patients with Chemotherapy-Refractory Biliary Tract Cancer: A Multicenter Open-Label Phase 1/2a Trial
Source: Cancers (Basel). 2022 Aug 30;14(17):4229. doi: 10.3390/cancers14174229 (PMC9454779; doi:10.3390/cancers14174229)
Supplement: Supplementary file 1 [file cancers-14-04229-s001.zip › Supplementary Figures.pdf]

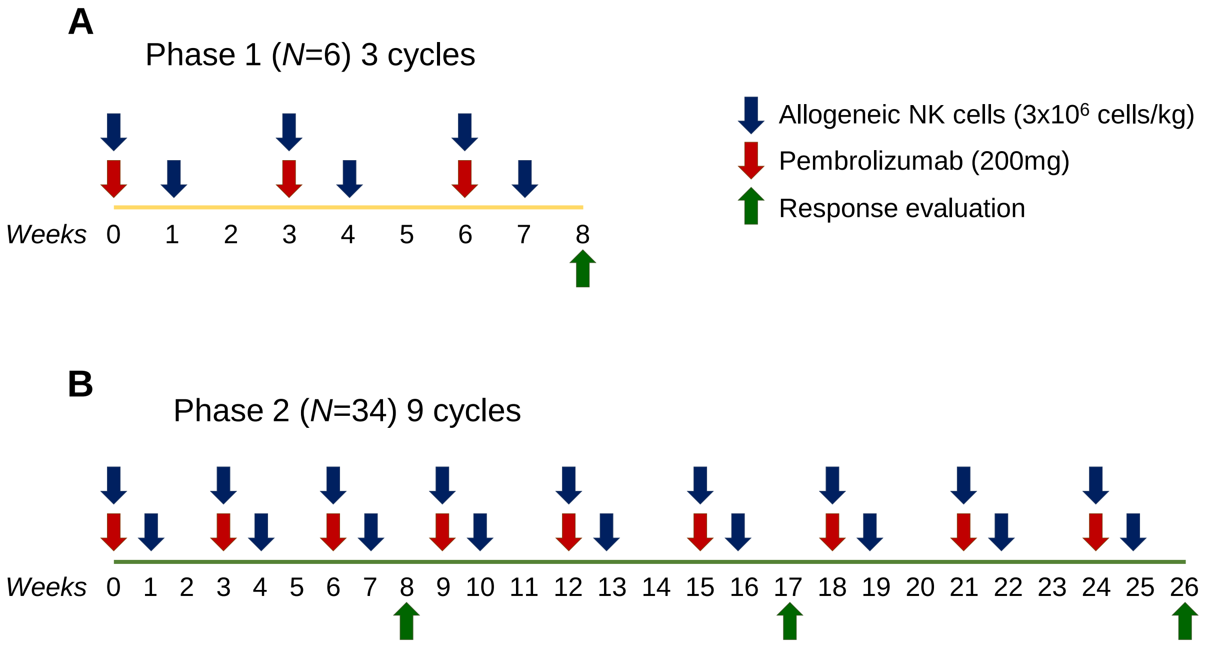


**Figure S1. Study protocol for the phase 1 (A) and phase 2a (B) trials.**

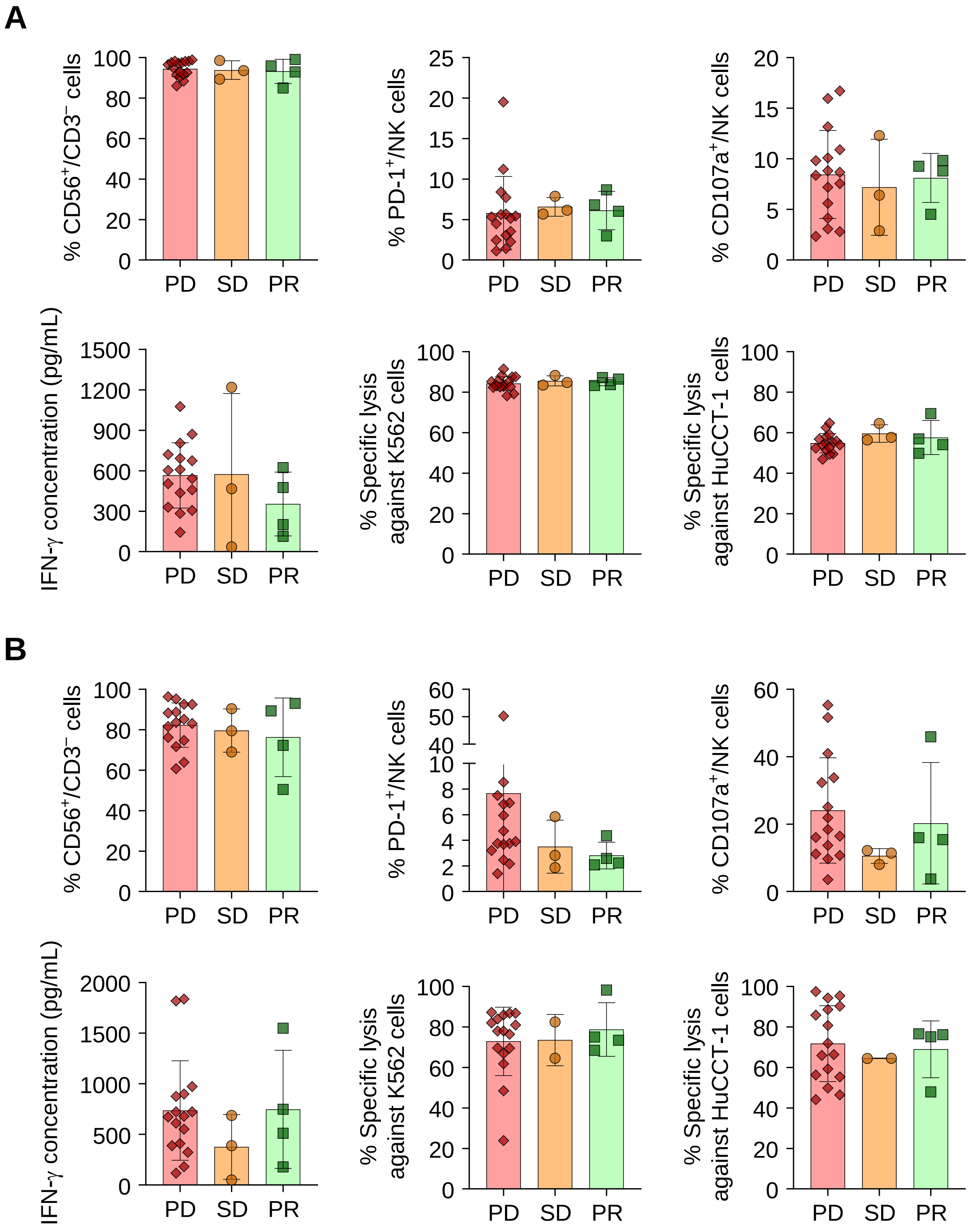


**Figure S2. The characteristics and functional activities of NK cells from donors (A) and recipients (B).** Cumulative data regarding the percentage of $CD56^{+}$ cells in $CD3^{-}$ cells, PD-1-expressing cells in NK cells, CD107a-expressing cells in NK cells, IFN-γ concentrations in the culture supernatants of isolated NK cells, NK cell cytotoxicity against K562 cells, and HuCCT-1 cells are presented. Red bars indicate the patients whose final tumor response was progressive disease (PD), orange bars indicate the patients whose final tumor response was stable disease (SD), and green bars indicate the patients whose final tumor response was a partial response (PR). Error bar indicates standard deviation (SD).
