# Supplementary material for: Safety and Efficacy of Allogeneic Natural Killer Cells in Combination with Pembrolizumab in Patients with Chemotherapy-Refractory Biliary Tract Cancer: A Multicenter Open-Label Phase 1/2a Trial
Source: Cancers (Basel). 2022 Aug 30;14(17):4229. doi: 10.3390/cancers14174229 (PMC9454779; doi:10.3390/cancers14174229)
Supplement: Supplementary file 1 [file cancers-14-04229-s001.zip › Supplementary Tables.pdf]

Table S1. Inclusion and exclusion criteria for patient enrollment

**Inclusion Criteria**
1. Age over 19 years
2. Histologically confirmed unresectable advanced biliary tract cancer who progressed after at least 1 regimen of chemotherapy or were unable to proceed with chemotherapy due to significant side effects
3. Eastern Cooperative Oncology Group (ECOG) Performance Status ≤ 2
4. Expected survival longer than 3 months
5. Laboratory tests fulfills following:
- Absolute neutrophil count (ANC) ≥ 1500 μL
- Hemoglobin ≥ 10 g/dL
- Platelet > 100,000/μL
- Blood urea nitrogen (BUN), Creatinine ≤ 1.5 X ULN
- Aspartate aminotransferase (AST), alanine aminotransferase (ALT) ≤ 2.5 X ULN
- Bilirubin ≤ 3 mg/L
6. Negative for pregnancy test
7. Fulfills one of the followings:
- [a]Combined positive score (CPS) of PD-L1 expression in tumor ≥ 1%
- Positive for [b]microsatellite instability (MSI)–high (H) or [c]deficient mismatch repair (dMMR)
**Exclusion Criteria**
1. Any history of immunodeficiency or autoimmune diseases (e.g., rheumatoid arthritis, systemic erythematous cyst, vasculitis, multiple sclerosis, Crohn's disease, ulcerative colitis, adolescent insulin-dependent diabetes, etc.) that can be exacerbated by immunotherapy
2. Treatment with systemic steroid therapy or anti-angiogenic agents
3. Pneumonia, colitis, hepatitis, nephritis, and endocrine diseases (hypophysitis, thyroid dysfunction, Type 1 diabetes, etc.) associated with immunodeficiency
4. History of other malignancy within 5 years
5. Treatment of conventional chemotherapy within 4 weeks
6. Severe neurologic or cardiac impairment
7. Severe allergic diseases
8. Serious mental illness
9. Pregnant women, lactating women, or a woman who intends to conceive during the trial
10. Enrolled in other clinical trials within 4 weeks
11. History of pembrolizumab treatment or other anti-PD-1 or anti-PD-L1 agents
12. History of NK cell therapy
13. Serious active infection

14. Inability to give informed consent

ULN; upper limit normal, NK; natural killer.

[a] CPS is examined using immunohistochemistry 22C3 pharmDx test, and calculated by dividing the number of PD-L1 positive tumor cells, lymphocytes, macrophage by total number of viable tumor cells, and multiplies 100.

[b] MSI-H was determined by polymerase chain reaction (PCR) methods and read positive when two or more unstable markers were found among 5 microsatellite markers.

[c] dMMR was analyzed by immunohistochemical (IHC) staining and read positive when one or more genes were lost in staining for MLH1, MSH2, MSH6, and PMS2.

Table S2. Inclusion and exclusion criteria for healthy NK cell donor

**Inclusion Criteria**
1. Age 19 to 69 years
2. Able to give informed consent and voluntarily agreed to participate in the scheduled blood tests and examination
**Exclusion Criteria**
1. Body weight < 45 kg
2. Systolic blood pressure < 90 mmHg or > 180 mmHg
3. Heart rate < 50/min or > 100/min
4. Body temperature > 37.5°C
5. Positive for any of the following tests in the blood tests:
- Hepatitis B surface antigen (HBs Ag)
- Hepatitis B virus nucleic acid amplification testing (HBV-NAT)
- Hepatitis C virus antibody (Anti-HCV)
- HCV-NAT
- Human immunodeficiency virus (HIV)
- HIV-NAT
- Syphilis rapid plasma regain (RPR) or fluorescent treponemal antibody absorption test (FTA-ABS)
- Human T-cell lymphotropic virus type 1/2 (HTLV-1/2)
- Cytomegalovirus antibody immunoglobulin M or immunoglobulin G (CMV-IgM or CMV-IgG)
-CMV-NAT

Table S3. Demographics and baseline characteristics of enrolled patients

	Phase 1	Phase 2a		
	Safety set (*N*=6)	Safety set (*N*=34)	Full-analysis set (*N*=23)	Per-protocol set (*N*=8)
**Age, years**	57 (56-70)	66 (39-84)	66 (39-77)	66 (39-77)
**Sex**				
Male	2 (33.3)	19 (55.9)	14 (60.9)	5 (62.5)
Female	4 (66.7)	15 (44.1)	9 (39.1)	3 (37.5)
**Weight, kg**	58.8 (54.0-81.5)	63.1 (41.4-93.9)	63.2 (44.9-93.9)	66.1 (44.9-93.9)
**Height, cm**	165.2 (150.1-171.7)	163.2 (148.2-183.5)	165.0 (151.7-183.5)	165.4 (152.0-178.9)
**CA 19-9, U/mL**	2960.2 (1021.4-9896.6)	110.5 (18.5-2307.3)	117 (15.1-1390.1)	245.5 (57.8-919.7)
**Tumor location**				
Intrahepatic bile duct	1 (16.7)	11 (32.4)	6 (26.1)	2 (25.0)
Extrahepatic bile duct	4 (66.7)	17 (50.0)	13 (56.5)	5 (62.5)
Gallbladder	1 (16.7)	6 (17.6)	4 (17.4)	1 (12.5)
**Prior chemotherapy**	6 (100.0)	34 (100.0)	23 (100.0)	8 (100.0)
Gemcitabine-based chemotherapy	5 (83.3)	34 (100.0)	23 (100.0)	8 (100.0)
Other regimens	5 (83.3)	16 (47.1)	10 (43.5)	2 (25.0)

Data are presented as median (IQR) or n (%).

Table S4. Objective Response Rate (ORR) by Combined Positive Score in Phase 2a

	CPS, Combined Positive Score			[a]p-value
	<5%	5~30%	≥30%	
[b]**<Full-analysis set>**				
Total number of subjects	10	7	5	
**Tumor response**				
CR	0 (0.0)	0 (0.0)	0 (0.0)	0.034
PR	1 (10.0)	0 (0.0)	2 (40.0)	
SD	0 (0.0)	3 (42.9)	0 (0.0)	
PD	9 (90.0)	4 (57.1)	3 (60.0)	
**Objective response rate (=CR+PR)**	1 (10.0)	0 (0.0)	2 (40.0)	0.140
[95% CI, %]	[0.2-44.5]	[0.0-41.0]	[5.3-85.3]	
**<Per-protocol set>**				
Total number of subjects	4	1	2	
**Tumor response**				
CR	0 (0.0)	0 (0.0)	0 (0.0)	0.114
PR	1 (25.0)	0 (0.0)	2 (100.0)	
SD	0 (0.0)	1 (100.0)	0 (0.0)	
PD	3 (75.0)	0 (0.0)	0 (0.0)	
**Objective response rate (=CR+PR)**	1 (25.0)	0 (0.0)	2 (100.0)	0.257
[95% CI, %]	[0.6-80.6]	[0.0-97.5]	[15.8-100.0]	

CR; complete response, PR; partial response, SD; stable disease, PD; progressive disease, CI; confidence interval.

[a] *P*-value for Log-rank test for the comparison among groups

[b] Pt E0107 who was positive for dMMR (PMS loss) was not included in this table.

Data are presented as n (%).

Table S5. Progression free survival (PFS) by Combined Positive Score in Phase 2a

	CPS, Combined Positive Score			[a]p-value
	<5%	5~30%	≥30%	
**[b]<Full-analysis set>**				
Total number of subjects	10	7	5	
**Median progression free survival (months)**	**3.0**	**3.9**	**4.6**	
[95% CI, %]	[1.7 – 5.9]	[1.6 – NA]	[3.7 – NA]	
**Number of subjects with PD or death**	9	5	3	0.546
[c]Hazard ratio [95% CI, %]	1	0.90<br>[0.30 – 2.70]	0.49<br>[0.13 – 1.82]	

NA; not available.

[a] *P*-value for Log-rank test for the comparison among groups.

[b] Pt E0107 who was positive for dMMR (PMS loss) was not included in this table.

[c] Hazard ratio is calculated by the proportional change in the hazard compared to the group of CPS < 5%.

Table S6. Adverse events by system organ class

System Organ Class<br>Adverse events	Phase 1 (*N*=6)		Phase 2a (*N*=34)	
	Any grade	Grade 3-5	Any grade	Grade 3-5
**Skin and subcutaneous tissue**				
Pruritus	1 (16.7)	0 (0.0)	5 (14.7)	1 (2.9)
Rash	1 (16.7)	0 (0.0)	0 (0.0)	0 (0.0)
**Gastrointestinal tract**				
Abdominal pain	0 (0.0)	0 (0.0)	4 (11.8)	1 (2.9)
Abdominal distension	1 (16.7)	0 (0.0)	2 (5.9)	0 (0.0)
Diarrhea	0 (0.0)	0 (0.0)	1 (2.9)	0 (0.0)
Ascites	0 (0.0)	0 (0.0)	2 (5.9)	1 (2.9)
Gastrointestinal bleeding	0 (0.0)	0 (0.0)	2 (5.9)	1 (2.9)
**Hepatobiliary**				
Jaundice	1 (16.7)	1 (16.7)	1 (2.9)	1 (2.9)
Acute cholangitis	0 (0.0)	0 (0.0)	1 (2.9)	1 (2.9)
Portal vein obstruction/stenosis	0 (0.0)	0 (0.0)	2 (5.9)	2 (5.9)
Liver abscess	1 (16.7)	1 (16.7)	0 (0.0)	0 (0.0)
**Infection**				
Viral infection	0 (0.0)	0 (0.0)	3 (8.8)	0 (0.0)
Sepsis	0 (0.0)	0 (0.0)	2 (5.9)	2 (5.9)
**Respiratory**				
Pleural effusion	0 (0.0)	0 (0.0)	3 (8.8)	0 (0.0)
Dyspnea	0 (0.0)	0 (0.0)	1 (2.9)	0 (0.0)
**Urinary tract**				
Acute kidney injury	1 (16.7)	1 (16.7)	2 (5.9)	1 (2.9)
**Cardiovascular**				
Hypotension	0 (0.0)	0 (0.0)	1 (2.9)	1 (2.9)
Arrhythmia	0 (0.0)	0 (0.0)	2 (5.9)	0 (0.0)
Ischemic heart disease	0 (0.0)	0 (0.0)	1 (2.9)	1 (2.9)
**Hematology**				
Anemia	0 (0.0)	0 (0.0)	4 (11.8)	3 (8.8)
Thrombocytopenia	0 (0.0)	0 (0.0)	2 (5.9)	1 (2.9)
Pancytopenia	0 (0.0)	0 (0.0)	1 (2.9)	1 (2.9)
**General**				
Fever	1 (16.7)	0 (0.0)	6 (17.6)	0 (0.0)
Headache	0 (0.0)	0 (0.0)	2 (5.9)	0 (0.0)
General weakness	0 (0.0)	0 (0.0)	5 (14.7)	0 (0.0)
Fatigue	0 (0.0)	0 (0.0)	2 (5.9)	0 (0.0)
Limb edema	0 (0.0)	0 (0.0)	3 (8.8)	0 (0.0)
Generalized edema	0 (0.0)	0 (0.0)	1 (2.9)	0 (0.0)

**Laboratory**				
Increased alkaline phosphatase	0 (0.0)	0 (0.0)	3 (8.8)	1 (2.9)
Increased gamma-glutamyl transferase	0 (0.0)	0 (0.0)	2 (5.9)	1 (2.9)
Decreased TSH	0 (0.0)	0 (0.0)	1 (2.9)	0 (0.0)

Data are presented as n (%).
